# Species conservation profile and amended distribution of *Cousinia knorringiae* (Asteraceae), a narrow endemic of the Western Tian-Shan

**DOI:** 10.3897/BDJ.9.e64115

**Published:** 2021-04-27

**Authors:** Mansur Usmonov, Komiljon Tojibaev, Chang-Gee Jang, Alexander N. Sennikov

**Affiliations:** 1 Kongju National University, Gongju, Chungnam, Republic of Korea Kongju National University Gongju, Chungnam Republic of Korea; 2 Institute of Botany, Academy of Sciences of the Republic of Uzbekistan, Tashkent, Uzbekistan Institute of Botany, Academy of Sciences of the Republic of Uzbekistan Tashkent Uzbekistan; 3 Finnish Museum of Natural History, University of Helsinki, Helsinki, Finland Finnish Museum of Natural History, University of Helsinki Helsinki Finland; 4 Komarov Botanical Institute, Saint-Petersburg, Russia Komarov Botanical Institute Saint-Petersburg Russia

**Keywords:** Central Asia, Compositae, conservation, East Fergana botanical hotspot, IUCN assessment, Kyrgyzstan, narrow endemic, new record, Uzbekistan

## Abstract

**Background:**

*Cousinia knorringiae* Bornm. (Asteraceae) belongs to C. sect. Subappendiculatae Tscherneva, a group of the species-rich and taxonomically difficult genus *Cousinia* Cass. This species is narrowly distributed in the Western Tian-Shan and has been known as endemic to Kyrgyzstan. It inhabits bare rocks and screes at elevations of 1200–1500 m above sea level. This species is of conservation interest because of its small population size and limited distribution.

**New information:**

*Cousinia knorringiae* is reported for the first time from eastern Uzbekistan on the basis of specimens collected on Ungur-Tepa Mt., a south-western outlier of the Bozbu-Too Mts. (Western Tian-Shan). The conservation status of the species is assessed as Endangered (EN), based on criterion D (estimated population size 200-250 mature individuals), according to the IUCN Red List Categories and Criteria (version 3.1). A new distribution map and a line drawing for *C. knorringiae* are provided and its type locality is clarified. The new knowledge suggests that the species is endemic to the East Fergana botanical hotspot, which includes a transborder territory shared between Kyrgyzstan and Uzbekistan and should, therefore, be subjected to international conservation measures. The southern extension of Ungur-Tepa Mt. harbours important plant species, which cannot be found elsewhere in Uzbekistan and may, therefore, be proposed for legal protection.

## Introduction

The mega-diverse genus *Cousinia* Cass. (Asteraceae) embraces about 600 species (Susanna and Garcia-Jacas 2006, López-Vinyallonga et al. 2009), many of which are closely related and very similar to each other. The species of *Cousinia* typically occur in arid territories, most commonly in the upland vegetation and are concentrated in mountainous areas of Turkey, Iran, Afghanistan and Central Asia (Knapp 1987). Central Asia is the main centre of its diversity; according to the classical revisions, ca. 260 species were recorded in the whole territory of Central Asia (Tscherneva 1993) and 133 species were known from Uzbekistan (Tscherneva 1962). In Uzbekistan, the species richness of *Cousinia* accounts for about 22% of the total diversity in Asteraceae and makes it the second largest genus of flowering plants after *Astragalus* L. (Tojibaev et al. 2014). Lately, eight species of *Cousinia* have been discovered in Uzbekistan (Sennikov 2010, Tojibaev et al. 2017, Usmonov 2017a, Usmonov 2017b), thus increasing its national diversity to 141 species. So far, this figure cannot be considered final and a comprehensive study of the genus in Uzbekistan is still required.

In the course of preparation of a taxonomic revision of *Cousinia* for the new *Flora of Uzbekistan* (Sennikov et al. 2016) and during the recent collecting activities in north-eastern Uzbekistan, we found a population of a previously unrecorded species of *Cousinia*, belonging to C. sect. Subappendiculatae Tscherneva. This section is characterised by wide, less numerous phyllaries and very finely attenuated apical anther appendages (Sennikov 2010). Having checked our specimens against the latest taxonomic revisions of *Cousinia* in Central Asia (Tscherneva 1993, Sennikov 2010), we identified the species as *C. knorringiae* Bornm., which has been known as a narrow endemic of Kyrgyzstan (Sennikov 2010, Lazkov and Umralina 2015, Tojibaev et al. 2020).

In the present contribution, we report the occurrence of *C. knorringiae* in Namangan Province of Uzbekistan, with images of collected specimens and plants in nature. Besides the new country record, we provide an updated distribution map, which is based on all known collections, and the first line drawing of the species. On the basis of the comprehensively collected distributional information, we assess a global conservation status of the species in the context of biodiversity hotspots in the Western Tian-Shan.

## Material and Methods

The new material for this study was collected in the field in Yangi-Kurgan District, Namangan Province, Uzbekistan in 2015. The vouchers were deposited at the National Herbarium of Uzbekistan, Institute of Botany (TASH).

Morphological characters were evaluated using identification keys and species descriptions (Tscherneva 1962, Tscherneva 1993, Sennikov 2010).

A distributional dataset was compiled on the basis of the published data (Sennikov 2010) with the addition of herbarium collections deposited at the Institute of Botany of the Academy of Sciences of Uzbekistan (TASH), Moscow State University (MW) and recent herbarium collections from the Institute of Biology and Soil Science of the Academy of Sciences of Kyrgyzstan (FRU) and the Botanical Museum, Finnish Museum of Natural History, University of Helsinki (H). Observations, documented by photographs (Anonymous 2020), were also taken into account. All specimens were re-examined *de visu* or from digital images. Specimens and observations were georeferenced with the help of printed maps adjusted with Google Maps. Coordinate uncertainty was assessed and uncertainly localised specimens were clustered with precisely documented records which were falling within the less accurately identified locations, in order to avoid duplication of locality information. Distributional maps were made using QGIS version 3.16.0 (QGIS Development Team 2020), with the base map produced as described in Lazkov and Sennikov (2017a).

Plants were photographed in the field in Uzbekistan and seed images were taken using an Olympus SZX12 stereo zoom binocular microscope, equipped with an eXcope XCAM1080 digital camera.

To assess the threat status of the species, IUCN Red List Categories and Criteria were employed (IUCN 2012). The geospatial analysis was performed using the Geospatial Conservation Assessment Web Tool GeoCAT (Bachman et al. 2011). The AOO was based on a recommended cell width of 2 km.

The textual data (plant description and assessments) and illustrations (photographs and drawings) were made publicly available through species pages in GBIF (Miller and Rycroft 2015). The distributional data were published through GBIF (Sennikov 2021).

## Results

We have revised all available collections and discovered 22 herbarium specimens and 2 observations of *Cousinia knorringiae* made to date. Altogether, the records were grouped into 10 localities, whose locations were recorded with the utmost precision available. The type locality was more precisely localised on the basis of available literature (von Knorring 1915). The recent collection from Uzbekistan makes a country record for the species. A restricted distribution area and a low number of individuals known suggest that the appropriate conservation status of *C. knorringiae* is Endangered (EN).

### Taxonomy and nomenclature

***Cousinia knorringiae*** Bornm., Beih. Bot. Centralbl. 34(2): 201. 1917 (Bornmüller 1917).

**Figure 1.**
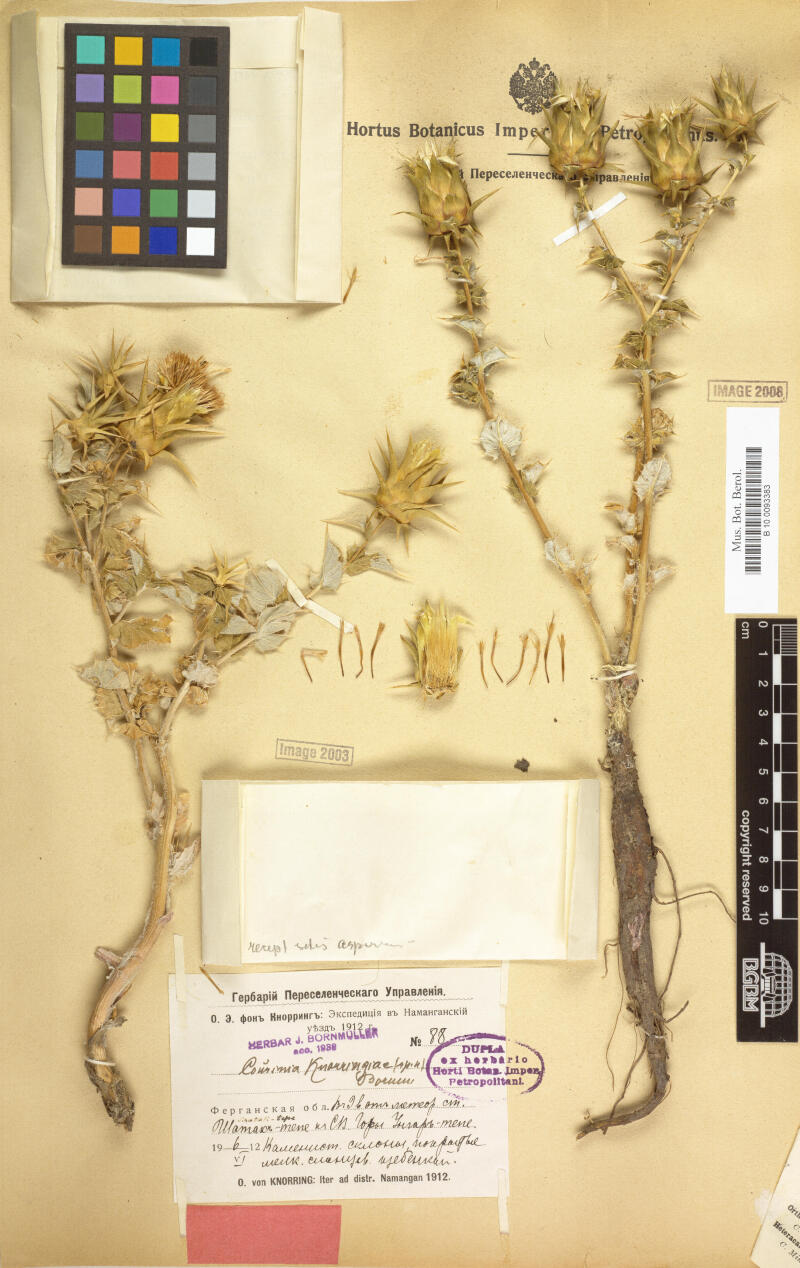
Lectotype specimen of *Cousinia knorringiae* (B barcode B100093383).

Type: Kyrgyzstan. “Fergana Valley, 9 versts NE of meteorological station Schatak-Tepe, Ungor-Tepa Mts.”, 06.06.1912, *O. von Knorring 88* (lectotype B barcode B100093383!, designated by Sennikov (2010); two isolectotypes LE!). Fig. 1.

**Figure 2.**
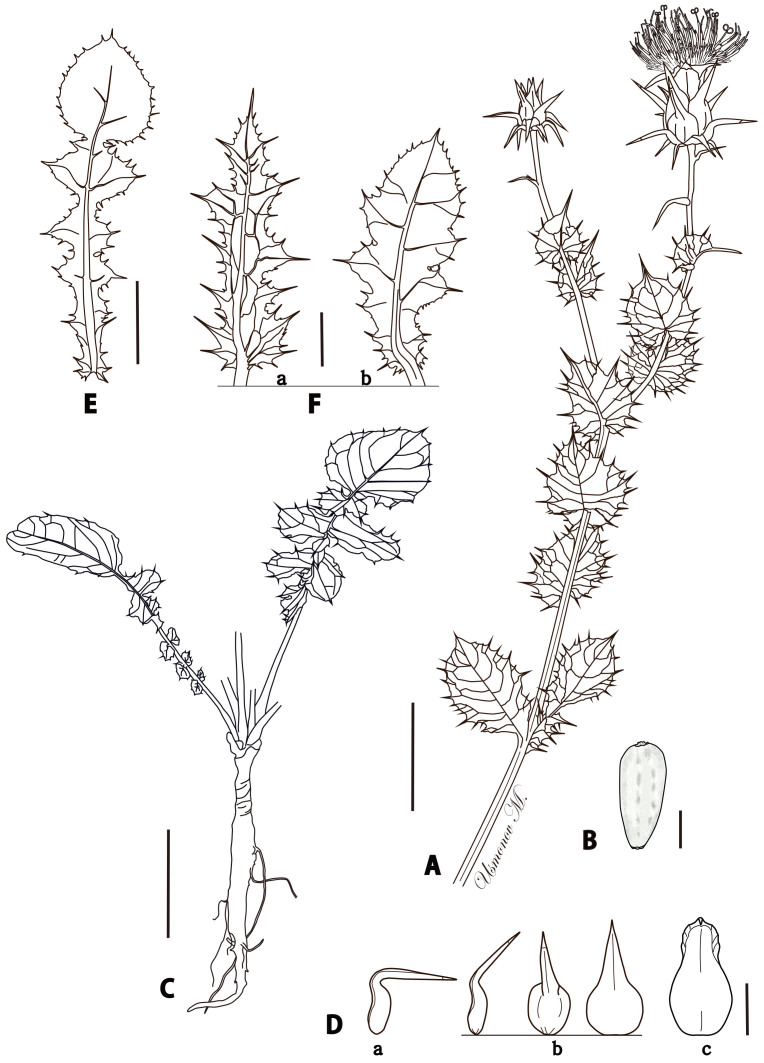
Line drawing of *Cousinia knorringiae*. **A**. Upper part of flowering stem; **B**. Seed; **C**. Rootstock with basal leaves; **D**. Phyllaries: **a**. outer phyllary (lateral view), **b**. middle phyllary (lateral and adaxial views), **c**. inner phyllary; **E**. Lower cauline leaf; **F**. Middle cauline leaves: **a**. oblong-lanceolate, **b**. oblong-ovate. Scale bars: A, C, E and F = 2 cm; B = 1 mm; D = 1 cm. Drawing: M. Usmonov.

Stems 40-50 cm high, very finely tomentose, branched from the base and above. Leaves coriaceous, tomentose slightly above and abundantly below, their nerves ending with spines up to 1 cm long; basal leaves up to 25 cm long, ca. 6 cm wide, lyrate with lanceolate-ovate upper lobe and up to 5 pairs of oblong-ovate lateral lobes; lower cauline leaves lyrate, withering at anthesis; middle cauline leaves 3.5-4.5 cm wide, oblong-ovate or obovate, incised at the lower half, round and amplexicaul at the base, with the auricles adnate to 0.2-0.5 cm, not decurrent; upper cauline ovate, amplexicaul with free auricles, long-spiny at the apex; uppermost not reduced. Capitula 1.5-2 cm broad (without appendages), its green part ca. 2 cm high. Phyllaries 25-35, dark-green, glabrous, hard-coriaceous, narrowly attenuated; outer phyllaries spreading horizontally or slightly downwards, some with 1-3 pairs of spines at the base, their reflexed part 6-12 mm long, 2 mm broad at the base, straight, narrowly triangular; middle phyllaries spreading obliquely upwards, their reflexed part 15-23 mm long, 5-7 mm wide at the base, folded and indistinctly contracted at the base, narrowly triangular, prominently concave; inner phyllaries appressed; innermost phyllaries with purple glabrous deltoid-lanceolate appendages 15-20 mm long, 3-5 mm wide, overtopping the green part of the involucre by 5-10 mm. Receptacular bristles scabrid. Corolla light-purple, prominently overtopping the involucre. Anthers stramineous, long and very narrowly attenuated at the apex. Achenes 4-4.5 mm long. Fig. 2.

Flowering period: May to June.

Distribution: Central Asia. Western Tian-Shan: Chatkal Mountain Range, Bozbu-Too Mts., At-Oinok Mts.; within the Kara-Suu River Basin. Kyrgyzstan, Uzbekistan.

Ecology: Gravelly slopes, rock crevices, open or partly shaded places, at elevations of 950-1500 m above sea level (optimal elevations between 1200 and 1400 m).

### Type locality

The type locality of *Cousinia knorringiae* is Ungur-Tepa Mt., which is situated on the border between Kyrgyzstan and Uzbekistan; the highest part and the southern foothills of the mountain belong to Uzbekistan, whereas its eastern, northern and western foothills belong to Kyrgyzstan. The type locality has not been precisely localised in earlier publications and its position did not appear on the otherwise accurate distribution map in Sennikov (2010). Due to the complicated position and shape of country borders in Central Asia, it was not fully certain from the territory of which country (Kyrgyzstan or Uzbekistan) the species was actually described.

**Figure 3.**
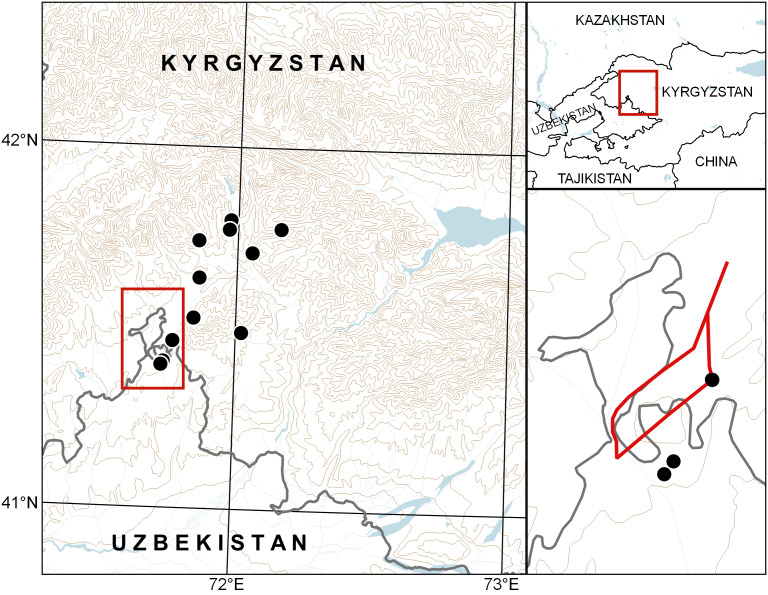
Distribution map of *Cousinia knorringiae*. Red line in the lower right part: route of the expedition of O. von Knorring and S. Neustruev across Ungur-Tepa Mt. in 1912.

According to the expedition route map, published by von Knorring (1915), she visited the territory of Ungur-Tepa Mt. while travelling from Turpak-Bel Pass to Zarkent Village and back. For the outward journey, Knorring went along the road around the northern side of the mountain and, on the way back, she crossed the mountain through its highest part (Fig. 3). While crossing the mountain, she passed the present-day territory of Uzbekistan only at the highest elevations, which are unsuitable for the species; the maximum height of Ungur-Tepa Mt. is 1956 m, whereas suitable habitats of the species are limited by the elevation of 1500 m (Sennikov 2010). From this fact, we safely conclude that Knorring collected the species on the foothills of Ungur-Tepa Mt., which she sampled in the territory of present-day Kyrgyzstan only and the species was, therefore, described from Kyrgyzstan.

The original label of Knorring refers to meteorological station "Shatak-tebe", or "Schatak-tebe" in contemporary German spelling, which historically existed at the very border with Uzbekistan (Cherkasov et al. 1987), on a small hill by the same name; the hill was described as composed of gravel and pebble overlaid with loesses (Neustruev and Nikitin 1926). This hill is apparently the smallest and farthest south-western outpost of Bozbu-Too Mts., adjacent to Ungur-Tepa Mt., which is situated in Kyrgyzstan at 41.394359°N, 71.625116°E and whose elevation is between 1250 and 1275 m. The hill is nameless on current maps. The exact position of this historical locality may be important in databasing historical records of plants and animals collected by the expedition of O. von Knorring and S. Neustruev in 1912, which brought a number of species new to science, described on the basis of their collections.

### New country record

**Figure 4.**
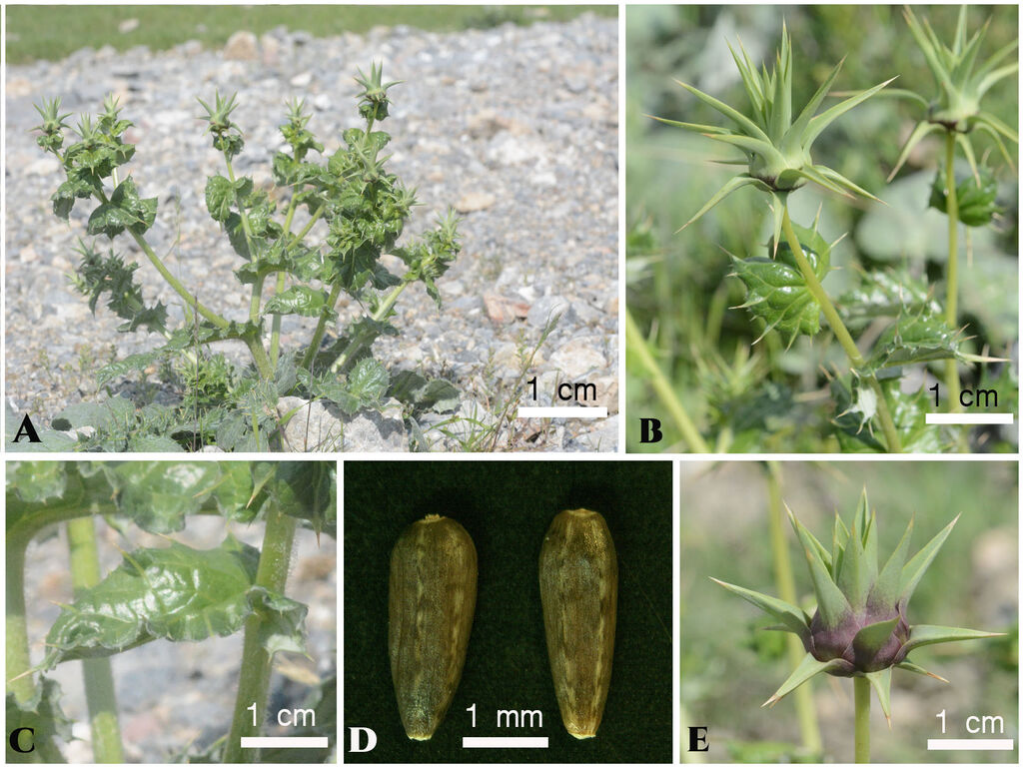
*Cousinia knorringiae* in Uzbekistan. **A**. Habit.; **B**. Immature capitula with upper cauline leaves; **C**. Middle cauline leaf; **D**. Seeds; **E**. Immature capitulum with red colouration. Photo: K. Tojibaev, except (d): M. Usmonov.

**Figure 5.**
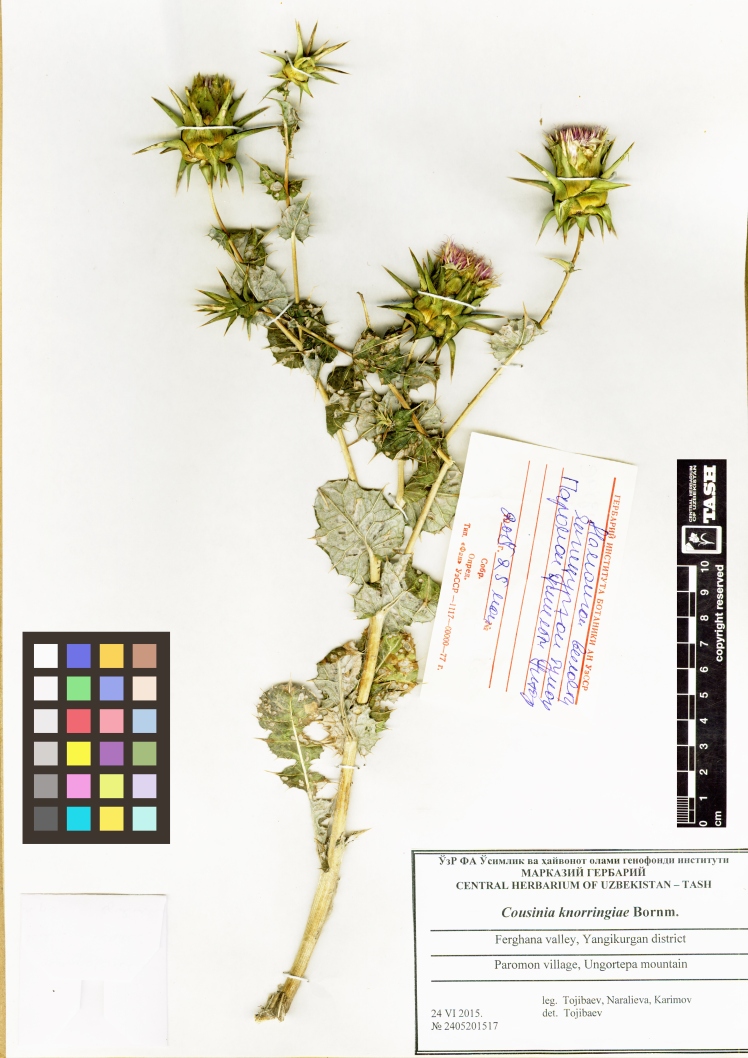
Representative specimen of *Cousinia knorringiae* from Uzbekistan (TASH).

According to the published data (Tscherneva 1993, Sennikov 2010, Lazkov and Sultanova 2014, Lazkov and Umralina 2015, Tojibaev et al. 2020), *Cousinia knorringiae* was considered endemic to Kyrgyzstan and our country record from Uzbekistan is, therefore, a novelty. The species (Fig. 4, Fig. 5) was discovered and collected by Tojibaev on the southern foothills of Ungur-Tepa Mt., north of Paromon Village, in Yangi-Kurgan District, Namangan Region, Uzbekistan (reference to GBIF).

### Distribution type

The species is endemic to the East Fergana botanical hotspot, the area known for a number of narrowly restricted plant species (Lazkov et al. 2002). This area includes the south-eastern parts of the Chatkal Mountain Range, Bozbu-Too Mts., At-Oinok Mts., Isfan-Jailoo Mts., Kyrbuu-Too Mts. and Babash-Ata Mts.; recent research continues adding new endemic plants described from this area (e.g. Sennikov and Lazkov 2013). The area lies almost entirely within Kyrgyzstan, with small outliers of mountain ranges shared with Uzbekistan. This share, although territorially minor, affects a number of narrowly distributed plant species which were previously considered endemics of Kyrgyzstan and, therefore, viewed as objects of national conservation concern (e.g. Lazkov and Sennikov 2017b). Recent knowledge suggests that transborder conservation efforts may be desirable in certain territories of the East Fergana botanical hotspot and Ungur-Tepa Mt. could be one of such territories, especially its southern extension with lower elevations (max. 1450 m) which is adjacent to Paromon Village. The southern extension of Ungur-Tepa Mt. harbours narrowly distributed plant species (e.g. *Allium dodecadontum* Vved., *Cousinia knorringiae* Bornm.) which cannot be found elsewhere in Uzbekistan and may, therefore, be proposed for legal protection at the national level with subsequent inclusion in transborder conservation networks.

## Species Conservation Profiles

### Cousinia knorringiae

#### Species information

Scientific name: Cousinia knorringiae

Species authority: Bornm.

Synonyms: No synonyms published.

Common names: No common names recorded.

Kingdom: Plantae

Class: 

Order: Asterales

Family: Asteraceae

Taxonomic notes: The species is placed in Cousinia sect. Subappendiculatae due to its longer coloured appendices of inner phyllaries and long-attenuated apical appendices of anthers (Sennikov 2010).

Figure(s) or Photo(s): Figs 2, 4

Region for assessment: Global

#### Editor & Reviewers

##### Reviewers

Reviewers: Lazkov, G.A. & Allen, D.J.

##### Editor

Editor: Sennikov, A.N.

#### Reviewers

Reviewers: Lazkov, G.A. & Allen, D.J.

#### Editor

Editor: Sennikov, A.N.

#### Geographic range

Biogeographic realm: Palearctic

Countries: UzbekistanKyrgyzstan

Map of records (image): Fig. 3

Map of records (Google Earth): Suppl. material 1

Basis of EOO and AOO: Observed

Basis (narrative): The species is known from 22 specimens kept at 6 herbaria worldwide (B, FRU, H, LE, MW, TASH) and 2 human observations, which document its occurrence in 10 localities separated by at least 2 km (Sennikov 2021). The best known locality is situated south of Arkyt, which has been sampled repeatedly for 70 years. The most recent locality is a new country record in Uzbekistan. The other localities have been sampled once or twice, largely in historical times.

Min Elevation/Depth (m): 950

Max Elevation/Depth (m): 1500

Range description: The species is mountainous; it grows in three mountain systems of the Western Tian-Shan in Central Asia: Chatkal Mountain Range (south-eastern part), Bozbu-Too Mts., At-Oinok Mts., within the Kara-Suu River Basin.In spite of its restricted distribution, the species occurs in the territory of two countries, Kyrgyzstan and Uzbekistan. The main distribution area lies within Kyrgyzstan, whereas only one locality is situated in Uzbekistan.

#### New occurrences

#### Extent of occurrence

EOO (km2): 850

Trend: Unknown

Justification for trend: Although the current species situation is stable, it is sensitive to habitat destruction by mining and road construction, which is very common in the Western Tian-Shan. So far, no extinction in any species locality has been documented and its complete distribution area is under research.

Causes ceased?: Unknown

Causes understood?: Unknown

Causes reversible?: Unknown

Extreme fluctuations?: Unknown

#### Area of occupancy

Trend: Unknown

Justification for trend: So far, no populations are known as extinct. Decline is possible because of the vulnerability of habitats, but the lack of monitoring data does not allow assessment of its extent.

Causes ceased?: Unknown

Causes understood?: Unknown

Causes reversible?: Unknown

Extreme fluctuations?: Unknown

AOO (km2): 44

#### Locations

Number of locations: 10

Justification for number of locations: The species occurrence is documented in 10 localities separated at least by 2 km. The best known locality is situated south of Arkyt, which has been sampled repeatedly for 70 years. The most recent locality is a new country record in Uzbekistan. The other localities have been sampled once or twice, largely in historical times.

Trend: Unknown

Extreme fluctuations?: Unknown

#### Population

Number of individuals: Estimated as fewer than 250 mature individuals.

Trend: Unknown

Justification for trend: With certainty, no expansion can be inferred. Based on continuous sampling and observations, the species may be considered rather stable. However, based on its poor reproduction and paucity of individuals in populations, the species may be sensitive to any external factors and some level of decline is likely.

Causes ceased?: Unknown

Causes understood?: Unknown

Causes reversible?: Unknown

Extreme fluctuations?: Unknown

Population Information (Narrative): The exact population size and the number of individuals are unknown. A population observed by A. Sennikov & G. Lazkov near Arkyt was visited repeatedly for nearly 70 years, but less than 20 mature individuals were counted with few immature individuals. According to G. Lazkov (pers. comm., 2020), this population can be considered typical of the species. A total of 30 mature individuals were observed in Uzbekistan (K. Tojibaev, pers. comm., 2020). Based on these observations, we estimate that the species may be represented by less than 250 mature individuals.Some biennial species of *Cousinia* in Kyrgyzstan may display population waves, nearly disappearing from sight in certain years (Sennikov, pers. obs.); however, this behaviour is unknown in *C. knorringiae*.

#### Subpopulations

Trend: Unknown

Extreme fluctuations?: No

Severe fragmentation?: No

#### Habitat

System: Terrestrial

Habitat specialist: Yes

Habitat (narrative): The species grows on gravelly slopes and in rock fissures, in open places, typically insolated or sometimes slightly shaded. It grows on bare rocks and does not form plant communities.

Trend in extent, area or quality?: Unknown

##### Habitat

Habitat importance: Major Importance

Habitats: 6. Rocky areas (e.g. inland cliffs, mountain peaks)

#### Habitat

Habitat importance: Major Importance

Habitats: 6. Rocky areas (e.g. inland cliffs, mountain peaks)

#### Ecology

Size: 40-50

Generation length (yr): 2

Dependency of single sp?: No

Ecology and traits (narrative): The plants are biennial, forming a sterile rosette in the first year and flowering in the second year. The plants are medium-sized spiny forbs, not attractive to cattle and other large herbivores.

#### Threats

Justification for threats: Mining and road construction are common in rocky areas of the Western Tian-Shan and represent potential threats; however, no localities of the species are currently under direct impact for this reason. Seed set is partly limited by larvae of the Tephritidae and by mould, but to a lesser extent (A. Sennikov, pers. obs.).

##### Threats

Threat type: Future

Threats: 3.2. Energy production & mining - Mining & quarrying4.1. Transportation & service corridors - Roads & railroads

##### Threats

Threat type: Ongoing

Threats: 8.2. Invasive and other problematic species, genes & diseases - Problematic native species/diseases

#### Threats

Threat type: Future

Threats: 3.2. Energy production & mining - Mining & quarrying4.1. Transportation & service corridors - Roads & railroads

#### Threats

Threat type: Ongoing

Threats: 8.2. Invasive and other problematic species, genes & diseases - Problematic native species/diseases

#### Conservation

Justification for conservation actions: The species is not included in national Red Lists. One population near Arkyt is situated within the Sary-Chelek Nature Reserve in Kyrgyzstan. No introduction to any botanical garden is known. Seeds have not been submitted to the Millennium Seed Bank or any similar institution.We suggest nation-level protection of the species in Kyrgyzstan and Uzbekistan. Besides, other protected areas may be established to contribute to the sustainability of the species and other narrowly endemic plants of East Fergana. In particular, the southern foothills of Ungur-Tepa may be protected in Uzbekistan.

##### Conservation actions

Conservation action type: In Place

Conservation actions: 1.1. Land/water protection - Site/area protection

##### Conservation actions

Conservation action type: Needed

Conservation actions: 1.1. Land/water protection - Site/area protection

#### Conservation actions

Conservation action type: In Place

Conservation actions: 1.1. Land/water protection - Site/area protection

#### Conservation actions

Conservation action type: Needed

Conservation actions: 1.1. Land/water protection - Site/area protection

#### Other

Justification for use and trade: No use or trade of the species has been registered.

##### Use and trade

Use type: National

Use and trade: 18. Unknown

##### Ecosystem services

Ecosystem service type: Less important

##### Research needed

Research needed: 1.2. Research - Population size, distribution & trends1.5. Research - Threats3.1. Monitoring - Population trends3.4. Monitoring - Habitat trends

Justification for research needed: The complete distribution area of the species requires verification; more localities and populations can be discovered. However, the species is known as endemic to the East Fergana botanical hotspot and it is highly unlikely that any new locality will be found far away from the current distribution area.Population size and dynamics should be researched to better assess the viability of the species and its conservation status. The exact population size may be even lower than currently estimated because most of the localities have not been revisited by botanists.Population dynamics may appear important because population waves are known in species of the same genus. So far, no such observations exist for the species.Mining and road construction are apparent projected threats to the species. Their actual impact on the species viability should be assessed in all localities.

#### Use and trade

Use type: National

Use and trade: 18. Unknown

#### Ecosystem services

Ecosystem service type: Less important

#### Research needed

Research needed: 1.2. Research - Population size, distribution & trends1.5. Research - Threats3.1. Monitoring - Population trends3.4. Monitoring - Habitat trends

Justification for research needed: The complete distribution area of the species requires verification; more localities and populations can be discovered. However, the species is known as endemic to the East Fergana botanical hotspot and it is highly unlikely that any new locality will be found far away from the current distribution area.Population size and dynamics should be researched to better assess the viability of the species and its conservation status. The exact population size may be even lower than currently estimated because most of the localities have not been revisited by botanists.Population dynamics may appear important because population waves are known in species of the same genus. So far, no such observations exist for the species.Mining and road construction are apparent projected threats to the species. Their actual impact on the species viability should be assessed in all localities.

#### Viability analysis

## Supplementary Material

759FCD57-C66F-50C4-9660-2EA32787185D 10.3897/BDJ.9.e64115.suppl1 Supplementary material 1 Distributional data for *Cousinia knorringiae* Data type occurrences Brief description Observation data based on the comprehensive revision of herbarium specimens and published human observations, as used in GeoCAT. File: oo_494360.kml https://binary.pensoft.net/file/494360 Sennikov, A.N.
